# U.S. college students’ perspectives on contraception and abortion post-*Dobbs*: the influence of socioeconomic privilege and gender inequity

**DOI:** 10.3389/fpubh.2023.1274154

**Published:** 2024-01-10

**Authors:** Emily S. Mann, Jessica A. McLennan, Kathleen Broussard

**Affiliations:** ^1^Department of Health Promotion, Education, and Behavior, Arnold School of Public Health, University of South Carolina, Columbia, SC, United States; ^2^Department of Women's and Gender Studies, College of Arts and Sciences, University of South Carolina, Columbia, SC, United States; ^3^South Carolina Honors College, University of South Carolina, Columbia, SC, United States; ^4^Department of Sociology, College of Arts and Sciences, University of South Carolina, Columbia, SC, United States

**Keywords:** contraception, abortion, college students, reproductive health, United States

## Abstract

This study examined college students’ perspectives about contraception and abortion in the context of the United States Supreme Court’s decision to eliminate the constitutional right to abortion in June 2022. Individual, semi-structured interviews were conducted between October 2022 and February 2023 with a convenience sample of 20 college students, ages 18–22, attending a public university in the southeastern United States. Qualitative data analysis revealed three main themes. First, most participants conveyed fear, dismay, and anger about the decision in Dobbs v. Jackson Women’s Health Organization to overturn Roe v. Wade and a few expressed concerns about potential restrictions on contraception. Second, women participants felt heightened pressure to continue or initiate use of a highly effective contraceptive method, with some lamenting inequitable experiences of the gendered contraceptive burden in their relationships with men. Third, when asked what they would do if they or their partner became pregnant while in college, most asserted they would seek abortion. Notably, participants assumed their socioeconomic advantages would ensure their or their partner’s access to abortion, regardless of growing restrictions. The findings illustrate that among a group of relatively privileged young adults, the Dobbs decision simultaneously compelled their increased vigilance regarding contraceptive use and conferred the perception that they would not be personally impacted should they need an abortion.

## Introduction

In June 2022, the United States Supreme Court overturned the federally protected right to abortion in the *Dobbs v. Jackson’s Women Health Organization* decision. Prior to *Dobbs*, all states in the U.S. were required to provide access to abortion at least until “fetal viability,” although states were allowed to enact obstacles, provided they did not impose an “undue burden” (1). Following the decision, most states controlled by conservative politicians, primarily in the South and Midwest, have eliminated or severely restricted abortion access (2). These abortion restrictions have far-reaching implications for pregnancy-capable people’s reproductive autonomy. Due to increased delays in obtaining abortion care, more people are being denied abortion, and surveilled and criminalized for activities during pregnancy (3). Further, abortion restrictions disproportionately impact people who are young, racially marginalized, and economically vulnerable (4–7). The *Dobbs* decision has also raised concerns about the right to contraception, which was initially established in *Griswold v. Connecticut* in 1965 using the same constitutional provision that supported the *Roe v. Wade* decision in 1973 (1).

Young adults, ages 18–24, have long been the focus of reproductive health research on contraception and abortion, largely because they have higher rates of unintended pregnancy and abortion and lower rates of contraceptive use than older age groups (8–12). Most of this research highlights the racial and socioeconomic disparities in rates of unintended pregnancy, unplanned births, and abortion, and their association with lower rates of contraceptive use, including inconsistent use and non-use (8, 13). While college students have higher rates of contraceptive use and are less likely to experience an unintended pregnancy than the general population of young adults (8, 11, 14), they are also more likely to have adverse sexual health experiences (e.g., sexual assault) due in part to the university environment (15–17). Further, evidence of barriers to accessing contraception and related reproductive health services in this population suggests unmet need (18, 19).

The limited qualitative research focused on college students and contraception has primarily focused on college women’s views about and use of long-acting reversible contraception (LARC), trust in healthcare providers, and discursive strategies for negotiating their own LARC use (or lack thereof) in an era of heightened LARC promotion (20–23). Findings indicate that college women lack knowledge about different LARC methods (21), express a range of positive and negative orientations toward LARC (22), and rely on neoliberal ideology to motivate their reasons for adopting or rejecting LARC (20). Less common are studies that include college men (24), in part because contraception is assumed to be a “women’s issue.” This pattern in the existing research reflects a structural form of gender inequality that Littlejohn (25) refers to as *gendered compulsory birth control*, whereby women of reproductive age are systemically expected to use prescription contraception (e.g., oral contraceptive pills) to prevent pregnancy. Compulsory contraceptive use is thus a burden that women alone are supposed to shoulder. In the face of mounting abortion restrictions post-*Dobbs*, women’s reproductive autonomy is not only constrained by laws that restrict their capacity to terminate a pregnancy if needed, but also the potential intensification of pressure to use highly effective forms of prescription contraception (i.e., LARC). Cumulatively, the existing scholarship focused on young adults, contraception, and gender inequity surrounding pregnancy prevention points to the need to better understand college students’ perspectives on contraception and abortion in the post-*Dobbs* era.

## Materials and methods

### Study design

The study draws on individual, semi-structured interviews conducted between October 2022 and February 2023 with a convenience sample of 20 college students attending a large, predominantly white public university located in the southern region of the United States. The region’s conservative politics is well-documented and while many of the surrounding states had implemented highly restrictive abortion laws following *Dobbs*, at the time of data collection, abortion was legal up to 20 weeks of pregnancy in the state where the participants were attending college. The interviews examined participants’ experiences with sex education, relationship history, current and past contraceptive use, knowledge and attitudes about the *Dobbs* decision, perceptions of the effects of abortion restrictions on their contraceptive use, and whether they would seek abortion should they or their partner become pregnant.

To be eligible to participate in an interview, individuals had to be between ages 18 and 24 and currently attending the university where the data were collected. They did not need to be using contraception at the time of the interview and they could be of any gender. Informed consent was obtained from the participants using a verbal assent procedure whereby the interviewer read participants an informed consent script prior to the beginning of the interview. Participants then verbally consented to participate in an interview and were provided with a copy of the consent script. The study was approved the University of South Carolina’s institutional review board.

### Study participants

The 20 participants ranged from 18 to 22 years old. Among the participants, 13 were women and seven were men. Thirteen participants were in-state college students while the remainder were from out-of-state (*n* = 7). Most participants indicated they were white (*n* = 17); the remainder identified as Black (*n* = 1) or biracial (*n* = 2). The majority were heterosexual (*n* = 15) while five identified with some other sexual orientation. Eleven participants were in a long-term intimate relationship, while 9 were either single or casually dating. Fifteen participants were using a female-centered prescription contraception method, five indicated they were using condoms only, and one said he was engaging in abstinence for his current contraceptive use (see Table 1).

**Table 1:** Demographic characteristics (*n* = 20).

Characteristic	*N*
*Mean age, years*	*20*
Race/ethnicity	
White	17
Black	1
Biracial	2
Gender	
Woman	13
Man	7
Sexual orientation	
Heterosexual	15
Bisexual	4
Asexual	1
Relationship status	
Currently in a relationship	11
Single	9
Current contraceptive use^1^	
Condoms	11
Oral contraceptive pill	6
Intrauterine device (IUD)	5
Implant	2
Ring	1
Other (e.g., abstinence, withdrawal)	8

^1^Some participants reported currently using multiple methods so numbers do not sum to 20.

### Data collection

We recruited participants using a digital flyer circulated via university email listservs, social media, and snowball sampling. Respondents then completed a brief anonymous survey hosted on Google Forms, which we used to collect information about respondents’ demographic characteristics, history of contraceptive use, and interest in participating in a confidential individual interview. We second then contacted eligible respondents via email and invited them to participate in an interview. While 119 people completed the survey, we only interviewed 20 respondents due to a low response rate to our interview invitations and no-shows. Nonetheless, thematic saturation was reached with these 20 interviews. The second author, a 22-year-old white woman college student, conducted the interviews via Zoom or over the phone, based on each participant’s preference. The interviews lasted an average of 25 min. To protect their confidentiality, participants chose their own pseudonyms, which are used in all reports of the study findings. To thank them for their time, participants received a $20 Amazon electronic gift card. Those who completed the survey but not an interview did not receive an incentive.

### Data analysis

The first and second authors conducted qualitative data analysis using Dedoose. We used a thematic approach wherein we initially derived deductive codes from the interview guide and developed inductive codes through an iterative process of constant comparison across emerging categories of analysis. Our coding process was informed by the extant literature and the following research questions: What are college students’ knowledge and attitudes about the U.S. Supreme Court’s decision in *Dobbs v. Jackson Women’s Health Organization*? How is *Dobbs* influencing college students’ contraceptive use? If the participant found out they or their partner were pregnant, what do they think they would do about the pregnancy? Here we highlight deductive codes focused on participants’ narratives about their knowledge of and attitudes about the *Dobbs* decision, the influence of the *Dobbs* decision on their contraceptive use, and whether they or their partner would desire an abortion if they experienced a pregnancy at this point in their lives. Additionally, two inductive codes emerged. One involved unequal gendered dynamics around contraceptive use and the other entailed participants’ perceived capacity access to abortion, which included participants’ assertions that they were confident they would have the resources needed to obtain an abortion. Our results reflect the patterns we identified in the data via thematic analysis.

## Results

Below we highlight major themes that emerged across participants’ responses to a series of questions related to the *Dobbs* decision that the interviewer asked during the last section of each interview. Notably, most participants articulated an accurate understanding of the Court’s decision. Further, most expressed vehement opposition to the elimination of federal protection for abortion. However, when queried about how they thought the decision impacted their own contraceptive use and access to abortion, participants’ responses revealed outrage and concern about the *Dobbs* decision, and a strong conviction that should they require an abortion, they would be able to obtain one. Additionally, among the women participants who were not using highly effective prescription contraception, they disclosed feeling pressure to initiate use. Therefore, while the unequal gendered burden of contraceptive use prior to *Dobbs* is well-documented (25–27), the decision and its cascade effects at the state level appear to be exacerbating this form of gender inequality for young women by further restricting their reproductive autonomy.

### Views about *Dobbs*

When the interviewer asked Madeline, a white woman who was using oral contraceptive pills, “What is your understanding of the Supreme Court’s decision to overturn *Roe v. Wade*?” she replied,

[T]he way that I understand it is that it is suddenly way easier for individual states to enact their own abortion laws that are contrary to sort of what *Roe v. Wade* had established on a federal level. It wasn't any sort of immediate change on a nationwide level as much as opening the door for different state governments to establish their own laws… I think it was a massive step backward for American women, for women in a developed country. It was really hard news to hear.

A few participants indicated awareness of the potential implications of *Dobbs* for other rights related to privacy, such as the right to marry a same-sex partner. George, a white man whose female partner was using oral contraceptive pills, expressed,

I think that it is terrifying. It seems that the Supreme Court has been going through these cycles where every 50 years they look back at cases they decided, and with *Roe v. Wade* being the law of the land, for 50 years, for it to be overturned and to allow states to decide the right of privacy of individuals, I think is a scary idea. Especially [since] I have a lot of friends now in college who are gay and they're thinking, what's next? And I think that there should be a universal right to abortion. And that's what my [home] state does have, thankfully. I wish that would continue into the South and that hopefully over time, we can reestablish those protections that *Roe v Wade* had established for 50 years. It's just crazy to me that we're going back in time.

Like Madeline and George, most participants conveyed fear and dismay about the decision; however, a few participants expressed anger when discussing their understanding of the Dobbs decision. For example, BG, a white woman who was using the implant, said,

From my understanding, it is trying to take away the right of a woman to be able to go and get an abortion on her free will. Makes me angry. It's scary, as a woman, knowing that that is something that very easily could be a part of my life and knowing that it would be taken away is very scary.

Kiki, a white woman who was using the ring, also did not mince words when she responded,

Personally, I think it's bullshit that the government thinks that they have a right to patient's privacy with their healthcare provider. This isn't a matter of abortion, this isn't a matter of getting rid of a fetus. This isn't a matter of reproductive rights. It is straight up a matter of the government thinks they have a conversation with you and your healthcare provider. And I think it's bullshit. It makes me so mad.

By contrast, Mary, a white woman who was using an IUD, felt confused and overwhelmed by the decision. While she wasn’t entirely sure what the decision legally meant, she nonetheless found it unjust.

I don't know, I don't really get into all of this. I know it's a huge topic for girls. I don't think it's fair for … My whole thing is I don't like how men are making the decision, I think that's absolutely super absurd, or just how they have any say. I don't know, it's very infuriating. I know the gist of it, I don't follow up with it, if that means anything….I think it's pretty unfair. I'm not really sure who's going to do something about that. I guess my whole thing with political things is my voice. I feel like it's so little that I don't know what to do with it, but I would hope somebody says that's super not right and do something about it.

Notably, Mary indicates awareness of how *Dobbs* reflects and reinforces gender inequity surrounding reproductive matters. At the same time, she does not see herself as someone who could take action to address the injustice of the decision. Instead, she hopes “somebody” else will “do something about it.”

### Influence of *Dobbs* on contraceptive use

After exploring participants’ understanding and interpretations of the *Dobbs* decision, the interviewer queried them on whether the decision was affecting their contraceptive use. Among the women participants already using LARC (*n* = 7), they expressed relief that they had the most effective form of reversible contraception available. For example, Katelyn, a white woman, said,

Well, it made me really glad that I got my IUD. I was kind of like, I'm very glad that I'm taking the right steps to prevent [pregnancy] even further. It solidified the fact that pregnancy feels a little unreversible. It's not unreversible, but it's a lot harder to reverse and it's terrifying, so it's just kind of reinforced that birth control is a beautiful thing that I want to be on.

Ella, a biracial woman, concurred, and elaborated,

Oh, I immediately was like, “Well, thank God I have an IUD,” because I know it can stay in for years because, God forbid, they make birth control illegal. And a lot of places are already pushing for that to happen, so I was like, “No way I'm taking this out, at least for now.” And as soon as I need a new one, if I'm in a place where I can get one, I will. Because the idea of that being in the air in the future is terrifying.

While BG previously demonstrated a clear understanding of the *Dobbs* ruling, later she indicated that she did not think abortion was legally available anymore in her state, even though at the time of data collection, abortion was legal up to 20 weeks. Despite this misinformation, BG conveyed a deep investment in continuing to use the implant to ensure she did not become pregnant.

I mean, I'm not even thinking about getting off of it. I definitely want to stay on it and that I need to stay on it because I feel like if it is being put mostly on me to protect myself, then I'm going to take those steps to make sure that something doesn't happen that I don't want to happen. And so definitely making sure that I am doing everything in my control, that that wouldn't have to be an option. And now that it's not an option, I definitely want to make sure that I'm doing what I can to protect myself from [pregnancy].

For the women participants who were using a non-LARC prescription method, *Dobbs* prompted them to consider switching to LARC. For example, Emmaline, a white woman using oral contraceptive pills, said,

I was considering switching to an IUD just because it was a more permanent solution. So, if the next thing to go was birth control, it's not like the government can be like, “Yes, you have to come in and have surgery to get that taken out.” Whereas for birth control pills, they could be like, “We're not filling your prescriptions anymore.” I did not end up doing that just because I think being from [home state] usually the decisions are a little less extreme than the ones in [current state], so I assumed that it would kind of work out okay. Which so far it has.

By contrast, Nathan, a white man in a relationship with a woman using oral contraceptive pills, said, “It has not really affected me at all, cause I’ve always used birth control and tried to prevent pregnancy, so I do not think it’s had any effect or change to my behavior or anything like that.” Despite reporting “always using birth control,” Nathan’s reliance on oral contraceptive pills indicated that his partner was primarily responsible for contraceptive use.

His lack of concern or change in behavior following the *Dobbs* ruling differed significantly from participants who had a physical capacity for pregnancy and themselves were managing their use of birth control.

Other women participants lamented the pressure they felt to continue or begin using more effective methods of birth control. Mary, a white woman who was using an IUD, said,

It just makes me even more upset that I'm on it. I feel like they have so little regard for me. My willingness to take birth control, it's just … I don't know, you're going to make me take it, but not help me if I did get pregnant or something?

Similarly, Riley, a white woman using condoms and fertility awareness, was concerned that she might have to use a hormonal contraceptive method.

It has made me reconsider if I want to go on hormonal birth control or not…As for now, I'm sure I will stick with the birth control that I'm currently using, but if I start to see, this is really becoming big, and a lot of states are now making [abortion] illegal…well then, it's in my hands now. Which kind of stinks because it turns you back onto that argument of who is responsible for birth control. And it's like, “well, now it seems like I am”…there's not really forms of birth control for men. And I think that puts a lot of pressure on women to make sure that they are on the right birth control and they're monitoring it constantly. It's another stress in our lives that I don't think should or needs to be there.

Together, Riley’s and Mary’s perspectives highlight how *Dobbs* and emerging state-level abortion restrictions can exacerbate *gendered compulsory birth control* (25). By restricting access to abortion, states participate in pressuring pregnancy-capable people to use specific methods of birth control when they would otherwise not prefer to use them.

### Perspectives on abortion post-*Dobbs*

At the end of each interview, the interviewer asked participants, “If you were to find out today that you (or your partner) were pregnant, what do you think you would do?” With a few exceptions, participants conveyed a strong desire to terminate the pregnancy. Mary reflected this pattern when she replied,

I would definitely probably get an abortion, or try to at least. I'm not ready for a child. Financially, no. Mentally, absolutely not. It's crazy, I can't even imagine my life with [a] child right now…There’s been conversations [with male partners]. It hasn’t been a genuine that’s what’s going to happen, but I feel like every person that I’ve been with, we’ve had that discussion if I would or not. They’re pretty much on the same page, if that would happen, they would agree.

While most people focused solely on how they would personally handle a hypothetical unintended pregnancy, Katelyn’s response recognized both her own privilege and inequities in abortion access,

I would try to terminate the pregnancy anyway that I possibly could. That’s not in my life plan right now…I think I’m privileged enough that I can handle it myself, that I could have friends who I could stay with in other states. So honestly, realistically for me, if I were to get pregnant in [current state], I still could find a way to access abortion. I would definitely choose to, honestly, but I would be able to access it and I would be able to get to an abortion center that was safe and relatively affordable for me. I think it’s kind of BS that a lot of people don’t have that option, they don’t have the ability to afford it or the ability to travel and there’s barriers now that exists that are not okay.

Kiki also perceived that her privilege would ensure her access to abortion, should she need one,

I live with the security that my parents have the money to fly me to another state. If I do need to get an abortion, my mom would be okay with me wanting to get an abortion. My boyfriend’s mom would also be okay with it. She actually has [different anglophone country] citizenship, so if shit hits the fan and I can’t get one in the U.S. I can go to [different anglophone country] …I would get an abortion. Definitely.

While a few female participants did express some uncertainty about whether they would seek an abortion should they become pregnant, most were unequivocal. Notably, among the men in the study, their responses highlighted their support for women’s reproductive autonomy, even if that might ultimately conflict with their own preferences. Penguin, a white man who was not in a relationship but reported using condoms, reacted to the question by saying,

God. I mean, I would hope that they would get an abortion, but I wouldn't pressure them. I'd say, “Let's talk about it. Let's see more options.” I don't think I could deal with a child right now just with I am so busy with everything going on. But also, it doesn't make sense why men should have a say in women's opinions in the matter. I guess these old white men, I mean, that's going to be me, but it's a woman's body. It's a woman's choice, I guess. But yeah, no, just God, my mom would probably think I was joking if I told her. But I would not want a kid. But it's up to the decision of the girl. But I'd do my best to be there for her, I guess.

Somewhat similarly, Nathan reflected,

That is a tough question. I'm not sure. I know [my girlfriend] personally disagrees with abortions. She's not pro-life in the sense that she wants them outlawed, but she says that her [*sic*] personally would not get an abortion because of her Catholic religious beliefs. But I'm not sure how that would all work out. I'm not sure how I would feel. I would probably want her to get an abortion, but I'm not going to make anybody do that, so we'd probably have to have a really serious long conversation about that.

Cumulatively, nearly all of the participants were confident they would have the resources to obtain an abortion. At the same time, most were preoccupied with ensuring that they would not need abortion access, provided they were able to access and consistently use highly effective contraception.

## Discussion

The study findings have multiple implications, which are both specific to college students and point to broader areas of concern regarding reproductive autonomy, gender inequities in contraceptive use, and collective action. The participants’ comprehension and criticism of the *Dobbs* decision indicate a fairly high level of political engagement in this population. This is not necessarily surprising, given studies demonstrating that undergraduate education increases political engagement and in turn, informs active participation in civic life (28). Further, emerging evidence from survey data point to this group’s strong support for abortion rights (29). Nonetheless, most participants indicated that they were heavily invested in using highly effective contraceptive methods to try to ensure they or their partner could avoid experiencing an unintended pregnancy.

Notably, some women participants pointed out the unequal gendered burden that fell on them to use female-centered prescription methods and indicated they felt external pressure to initiate using a more effective method as a result of *Dobbs.* While some acknowledge that the state was imposing these contraceptive burdens through abortion bans, most did not recognize that this was an interpersonal problem as well. Instead, most participants, regardless of gender, seemed to take for granted that sexually active women must use prescription contraception to prevent pregnancy. These dynamics point to the ways abortion restrictions exacerbate prevailing gender inequities regarding pregnancy-capable people’s capacity to choose abortion and to make autonomous decisions about contraceptive use that reflect their own preferences and needs.

While participants expressed strong feelings when articulating their opposition to the *Dobbs* decision, collectively they did not indicate any motivation to engage in political action that would convey that opposition or seek change that might expand protections for abortion. It is possible this seeming complacency was due to their shared perception that they personally would be able to access abortion, if necessary. Participants’ sense that they would always be insulated from the direct consequences of *Dobbs* due to their socioeconomic privilege stood in contrast to their awareness of the possibility of a nationwide abortion ban and threats to contraception access. This disconnect between their political views and actions points to an opportunity for mobilization by the reproductive justice movement. Interest convergence (30) is needed, however, to puncture the apparent naïveté among some participants regarding the state’s capacity to infringe on their right to bodily autonomy, despite their advantages.

Interpretation of our findings must account for the limitations of the study design. We relied on a convenience sample of college students attending a large public university in the southeastern United States that was racially homogenous (e.g., predominately white) and high socioeconomic status; therefore, our findings cannot be construed to represent the views and experiences of U.S. college students generally. Nonetheless, a majority of the participants indicated that they would seek abortion if they or their partner became pregnant during this period of their lives. Previous research finds that abortion is significantly under-reported in survey research (31). By contrast, this prospective question about abortion decision-making elicited affirmative responses and revealed that some participants had determined (often in conversation with their partners) that abortion would be their preferred option in the case of an unintended pregnancy while in college. Many had even considered how they would obtain care in the face of legal restrictions in the region where their university was located. We are unable to determine whether these patterns are a result of the selective sample, the interviewing method, or the prospective (vs. retrospective) nature of the question. Future research should consider these differences to measure potential interest in and need for abortion care at the population level, particularly in states where abortion is banned or otherwise restricted.

This study also adds to the small but growing body of research on college student’s attitudes, perspectives, and decision-making around the use of LARC. While other studies have documented pressures to use LARC from healthcare providers and intimate partners (25, 27, 32), our analysis highlights how the U.S. state at both the federal and state levels can essentially pressure young women into using prescription contraceptive methods and unequal gendered compulsory contraceptive use as a result of the *Dobbs* decision. Further, we interviewed college students within the first year that *Roe v. Wade* was overturned, a decision that is dramatically curtailing reproductive autonomy; as our findings reveal, *Dobbs* is shaping people’s perspectives and behaviors related to their contraceptive use. Although participants expressed anger and outrage at the decision, they often described how their individual contraceptive decisions and behaviors would protect them from needing an abortion. Those who were using LARC and hormonal methods were “grateful” for this protection, while those using other methods felt pressure to change to methods that were more effective at preventing pregnancy. Recent data shows that 59% of people who obtained abortion in the United States prior to *Dobbs* had completed at least some college (25% were college graduates) (33), suggesting that college attendance does not shield people from needing abortion care. Reflecting either their socioeconomic privilege or naïveté about current and future abortion restrictions, as well as access to contraception, most participants we interviewed assumed they would be able to obtain abortion care if they needed it. Current research efforts are under way to document people’s ability to access abortion post-*Dobbs*, which will aid in expanding our understanding of the role of social class, including educational attainment, in access to care. While we await the results of these studies, we encourage reproductive justice advocates to focus on targeting U.S. college students for political mobilization against mounting legal constraints on pregnancy-capable people’s reproductive autonomy.

## Data availability statement

The datasets presented in this article are not readily available because of the sensitive nature of the interview data. Enquiries about this data should be directed to EM, emily.mann@sc.

## Ethics statement

The studies involving humans were approved by the University of South Carolina Institutional Review Board. The studies were conducted in accordance with the local legislation and institutional requirements. Written informed consent to participate in this study was not required from the participants in accordance with the national legislation and the institutional requirements.

## Author contributions

EM: Conceptualization, Formal analysis, Methodology, Supervision, Writing – original draft, Funding acquisition, Project administration. JM: Conceptualization, Methodology, Writing – review & editing, Funding acquisition. KB: Supervision, Writing – review & editing.
